# Three new species of *Conlarium* from sugarcane rhizosphere in southern China

**DOI:** 10.3897/mycokeys.56.35857

**Published:** 2019-07-05

**Authors:** Ling Xie, Yan-Lu Chen, Yan-Yan Long, Yan Zhang, Shi-Tong Liao, Bin Liu, Li-Ping Qin, Qian Nong, Wen-Long Zhang

**Affiliations:** 1 Microbiology Research Institute, Guangxi Academy of Agricultural Science, Nanning, Guangxi Province 530007, China Agricultural College of Guangxi University Nanning China; 2 Institute of Applied Microbiology, Agricultural College of Guangxi University, Nanning, Guangxi Province 530005, China Microbiology Research Institute, Guangxi Academy of Agricultural Science Nanning China

**Keywords:** Conlariaceae, conidial fungi, phylogeny, Rhizosphere, taxonomy

## Abstract

Three new species isolated from sugarcane rhizosphere in China, namely *Conlariumbaiseense***sp. nov.**, *C.nanningense***sp. nov.**, and *C.sacchari***sp. nov.**, are described and illustrated. Molecular evidence (phylogenetic analysis of combined LSU, SSU, ITS and RPB2 sequence data) and phenotypical characters support their independent status from related and similar species. The new species, as dark spetate endophytes, inhabit sugarcane rhizosphere and can form a symbiosis with sugarcane.

## Introduction

The genus *Conlarium*, described by Liu et al. (2012), belongs to the Conlariaceae, a family of freshwater ascomycetes (Zhang et al. 2017). This genus includes three species: *C.duplumascospora*, *C.aquaticum*, and *C.thailandense*. In these species, *C.duplumascospora* and *C.aquaticum* were isolated from submerged woody samples in streams (Liu et al. 2012; Zhang et al. 2017) and *C.thailandense* was isolated from dead wood (Phookamsak et al. 2019). During our ongoing survey of dark septate endophytes which inhabit sugarcane rhizosphere in Guangxi province, China, three undescribed species with the morphological characteristics of the genus *Conlarium* were isolated by the baiting method. The specimens were deposited in the Mycological Herbarium, Institute of Microbiology, Chinese Academy of Sciences, Beijing, China (HMAS).

## Materials and methods

### Fungal isolation and morphological studies

All soil samples were collected from the 5–15 cm deep sugarcane rhizosphere by five sampling methods in Guangxi province, China. Fungal isolations were obtained by using Chinese cabbage as a baiting plant, as described by Narisawa et al. (1998). Cultural characteristics were recorded after two weeks from potato dextrose agar (PDA). Conidiophores, conidiogenous cells, and conidia were examined as slide fungal preparations mounted in PVLG (polyvinyl alcohol, Lactic acid, Glycerin, and MiliQ water). Observations and measurements were made with Olympus BX53 Ci-L light microscope. Scanning electron microscopy (SEM) used a Tescan-vega3 LMU SEM.

### Molecular sequencing and phylogenetic analysis

The genomic DNA was extracted from mycelium grown on PDB (potato dextrose broth) at 28 °C for 10 d using the Prepman Ultra Sample Preparation Reagent Protocol (Applied Biosystems, California, USA). The large subunit ribosomal RNA gene (LSU), the small subunit ribosomal RNA gene (SSU), the internal transcribed spacer (ITS) rDNA, and the RNA polymerase II subunit 2 (RPB2) were amplified with fungal specific primers LROR/LR5, NS1/NS4, ITS1/ITS4, and fRPB2-5f/fRPB2-7cR (Vilgalys and Hester 1990; White et al. 1990; Liu et al. 1999). The PCR reaction mixture and conditions followed the modified protocol of 2×EasyTaq PCR SuperMix (TransGen Biotech, Beijing, China). Amplification was performed in a 50 μL reaction volume which contained PCR buffer [20 mM KCl, 10 mM (NH_4_)_2_SO_4_, 2 mM MgCl_2_, 20 mM Tris-HCl, pH8.4], 200 μM of each deoxyri-bonucleotide triphosphate, 15 pmols of each primer, 100 ng template DNA, and 2.5 units of Taq DNA polymerase (Biocolor BioScience and Technology, Shanghai, China). The thermal cycling program was as follows: 5 min initial denaturation at 94 °C, followed by 35 cycles of 40 s denaturation at 94 °C, 40 s annealing at 56 °C, 60 s extension at 72 °C, and a final 10 min extension at 72 °C. A negative control using sterilized distilled water instead of template DNA was included in the amplification process. The PCR products were examined by electrophoresis at 75 V for 2 h in 0.8 % (W/V) agarose gel in 1×TAE buffer (0.4 M Tris, 50 mM NaOAc, 10 mM EDTA, pH 7.8) and visualized under ultraviolet light after staining with ethidium bromide (0.5 μg ml^–1^) . The PCR products were purified using PCR Cleanup Filter Plates (MultiScreen ® PCRμ96; Millipore, USA) according to the manufacturer’s protocol. Purified PCR products were directly sequenced with primer pairs, as mentioned above, in an ABI 3730-XL DNA sequencer (Applied Biosystems, USA). The sequences were deposited at GenBank (http://www.ncbi.nlm.nih.gov) and compared in BLAST. Four kinds of rDNA sequences together with reference sequences (Table 1) were respectively aligned by MEGA v. 6.0 based on the neighbor-joining analyses and 1000 bootstrap replications.

**Table 1.** Taxa with GenBank accession numbers for SSU, ITS, LSU and RPB2.

**Taxa**	**Voucher**	**GenBank no.**			
		**SSU**	**ITS**	**LSU**	**RPB2**
* Conlarium nanningense *	M1	KX886203	KX886204	KX886202	MK224589
* Conlarium baiseense *	TD2	MF083159	MF083157	MF083158	MK573000
* Conlarium sacchari *	NN1	MF083162	MF083160	MF083161	MK224588
* Conlarium sacchari *	LA3	MF083165	MF083163	MF083164	MK573001
* Conlarium sacchari *	DX4	MF083168	MF083166	MF083167	MK224587
* Conlarium baiseense *	TD17	MK164657	MK164653	MK164655	MK572999
* Conlarium nanningense *	M8	MK164658	MK164654	MK164656	MK572998
* Conlarium duplumascospora *	CGMCC 14938	JN936987	JN936995	JN936991	NS
* Conlarium duplumascospora *	CGMCC 14939	JN936988	JN936996	JN936992	NS
* Conlarium duplumascospora *	CGMCC 14940	JN936989	JN936997	JN936993	NS
* Conlarium aquaticum *	MFLUCC 15-0992	MF374372	MF374354	MF374363	NS
* Conlarium thailandense *	MFLUCC 17-2349	MH624128	MH624129	MH624127	NS
* Atractospora thailandensis *	KUMCC 16-0067	MF374371	MF374353	MF374362	MF370951
* Atractospora reticulata *	CBS 127884	NS	KT991669	KT991660	KT991649
* Atractospora reticulata *	CBS 138740	NS	KT991670	KT991661	KT991650
* Atractospora decumbens *	CBS 139032	KT991640	KT991667	KT991658	KT991647
* Atractospora verruculosa *	CBS 132040	KT991641	KT991668	KT991659	KT991648
* Pseudoproboscispora thailandensis *	MFLUCC 15-0989	MF374377	MF374360	MF374369	NS
* Rubellisphaeria abscondita *	CBS 132078	KT991646	KT991678	KT991666	KT991657
* Lentomitella cirrhosa *	ICMP 15131	AY761089	KY931780	AY761085	KM492911
* Torrentispora biatriispora *	A 464-3	NS	KY931803	AY316352	KY931858

Notes: NS No data in GenBank.

Bayesian analyses of the same aligned four kinds of rDNA sequences dataset were conducted with MrBayes v. 3.1.2 (Huelsenbeck and Ronquist 2001) following the protocol of Sun and Guo (2010). The best-fit evolutionary model was determined for each dataset by comparing different evolutionary models via MrModeltest v. 2.3 (Nylander 2008). Four simultaneous chains of Markov Chain Monte Carlo were run starting from random trees and sampling every 100 generations. The analyses were halted at 4,000,000 generations for four kinds of rDNA sequences, when the calculation reached stationarity. At the end of the analysis, 4,000 trees were generated, respectively, and 25 % of them were excluded as the “burn in” when calculating the posterior probabilities. Bayesian posterior probabilities were obtained from the 50% majority rule consensus trees that remained. If more than 95% of the sampled trees contained a given clade, it was considered to be significantly supported by our data.

## Results

### Taxonomy

#### Conlarium nanningense

Taxon classificationFungiAnnulatascalesAnnulatascaceae

L.Xie, Y.L.Chen & B.Liu sp. nov.

MB821416

Figure 1

##### Etymology.

The species is named for Nanning City, the type locality.

##### Type.

CHINA. Guangxi: Nanning City, Datang Town. 22°23'25"N, 108°23'12"E, 144 m alt., in sugarcane rhizosphere, 11 Feb. 2011, L. Xie, M1 (HMAS 247075 holotype) deposited in Microbiology Research Institute, Guangxi Academy of Agricultural Science.

##### Description.

Colony reached 22 mm diameter on PDA medium after 2 weeks, grey-white to grey-brown, nearly circular, flat growth, less aerial hyphae. Hyphae grey-brown, verruculose, septate. Conidiophores 1–15 × 1–5 μm (6 ± 3 × 4 ± 1 µm, *n* = 54), stubby, unbranched, septate or aseptate, straight or flexuous, hyaline, becoming brown with age. Conidiogenous cells determinate, doliiform, cylindrical, 4–13 × 5–10 µm (6 ± 2 × 7 ± 2 µm, *n* = 22). Conidia brown, muriform, irregularly globose or subglobose, smooth, constricted at the septa, 0–1 transversely septa, 0–4 longitudinal septa, 11–21 × 9–21 µm (15 ± 3 × 13 ± 3 µm, *n* = 50). Chlamydospores subglobose or irregular, 4–12 µm (7±2 µm, *n* = 67). **Sexual morph**: undetermined.

**Figure 1.**
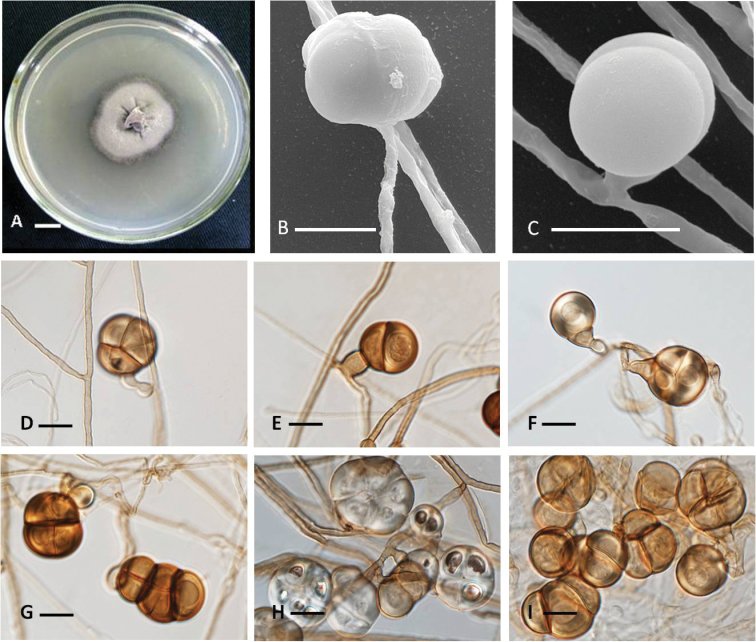
The new species *Conlariumnanningense* (HMAS 247075, holotype). **A** Colony morphology **B, C** Scanning electron microscopy of conidia **D–I** Mature conidia. Scale bars: 10 mm (**A**); 10 μm (**B–E**).

##### Habitat and distribution.

In sugarcane rhizosphere soil of southern China.

##### Other specimens examined.

CHINA. Guangxi: Nanning City, Datang Town. 22°29'54.51"N, 108°24'3.06"E, 102 m alt., in sugarcane rhizosphere, 11 Feb. 2011, L. Xie, M8 **(HMAS 247985).**

##### Notes.

*Conlariumnanningense* is similar to the asexual morph of *C.aquaticum*, *C.duplumascospora*, and *C.thailandense*. They all have monoblastic, holoblastic conidiogenous cells and mostly irregular, brown, clathrate, muriform conidia (Liu et al. 2012). However, *C.nanningense* can be easily distinguished from *C.aquaticum*, *C.duplumascospora*, and *C.thailandense* by the number of conidial septa (2–4-transversely septate, 1–3-longitudinally septate in *C.duplumascospora*; 6–12-transverse septa, 4–10-longitudinal septa in *C.aquaticum*; 4–8-transverse septa, 4–6-longitudinal septa in *C.thailandense* vs 0–1 transversely septa, 0–4 longitudinal septa in *C.nanningense*) and conidial size (15.5–35 × 11–26.5 μm in *C.duplumascosporum*, 45–70 × 20–57 μm in *C.aquaticum*, 25–45 × 17–33 μm in *C.thailandense* and 11–21 × 9–21 μm in *C.nanningense*) (Liu et al 2012; Zhang et al. 2017; Phookamsak et al. 2019). Phylogenetic reconstructions based on SSU+ITS+LSU+RBP2 sequences show authentic *C.nanningense* is sister to *C.duplumascospora*. A comparison of ITS pairwise indicates that *C.nanningense* differs from *C.aquaticum*, *C.duplumascospora*, and *C.thailandense* in 21 bp, 12 bp, and 18 bp, respectively. Thus, following the guidelines of Jeewon and Hyde (2016), this is a new species.

#### Conlarium baiseense

Taxon classificationFungiAnnulatascalesAnnulatascaceae

L.Xie, Y.L.Chen & B.Liu sp. nov.

MB821682

Figure 2

##### Etymology.

The species is named for Baise City, the type locality.

##### Type.

CHINA. Guangxi: Baise City, Tiandong County, Silin Town. 23°30'38"N, 107°20'1"E, 109 m alt., in sugarcane rhizosphere, 11 Sep 2015, Y.L. Chen and L.P. Qin, TD2 (HMAS 247298, holotype) deposited in Microbiology Research Institute, Guangxi Academy of Agricultural Science.

##### Description.

Colony reached 14 mm diameter on medium after 2 weeks at 28 °C, grey-white to grey, circular, flat growth, less aerial hyphae, regular edge of colony. Hyphae light yellow-green to light yellow-brown, septate. Conidiophores yellow-brown, mostly stubby, 0–2-branched, 0–8-septate, straight or flexuous, 3–12 × 2–6 μm (7 ± 2 × 4 ± 1 μm, *n* = 51). Conidiogenous cells determinate, doliiform, yellowbrown to brown, 3–8 × 5–12 μm (6 ± 1 × 7 ± 2 μm, *n* = 51). Muriform conidia yellow-brown to brown, irregularly globose or subglobose, smooth, constricted at the separation, 0–1 transversely septa, 0–4 longitudinal septa, 15–25 × 12–19 μm (18 ± 2 × 15 ± 2 μm, *n* = 26). Columnar conidia, yellow-brown to brown, 2–5 transversely septa, no longitudinal septa, 21–35 × 7–12 μm (28 ± 5 × 10 ± 1 μm, *n* = 23). **Sexual morph**: undetermined.

**Figure 2.**
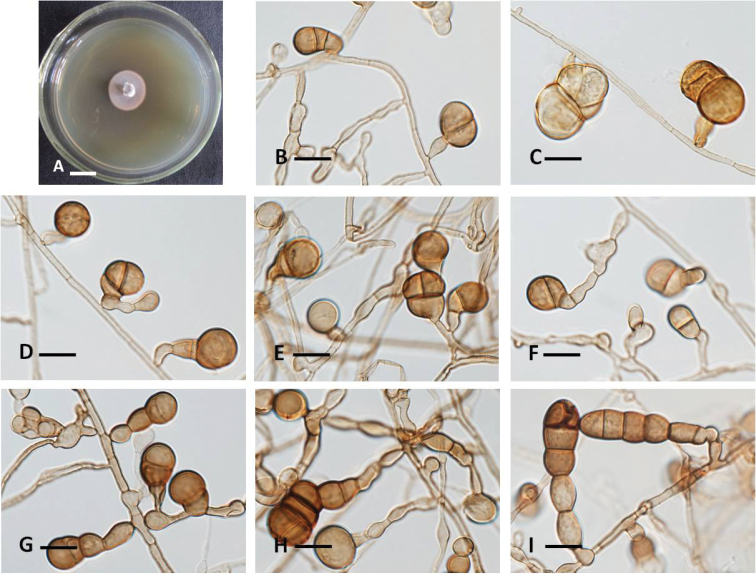
The new species *Conlariumbaiseense* (HMAS 247298, holotype). **A** Colony morphology **B–I** Conidiophores, conidiogenous cells and conidia. Scale bars: 10 mm (**A**); 10 μm (**B**).

##### Habitat and distribution.

In sugarcane rhizosphere soil of southern China.

##### Other specimens examined.

CHINA. Guangxi: Baise City, Tiandong County, Silin Town. 23°30'3.68"N, 107°20'1"E, 112.5 m alt., in sugarcane rhizosphere, 11 Sep. 2015, Y.L. Chen and L.P. Qin, TD17 **(HMAS 247986)**.

##### Notes.

*Conlariumbaiseense* is similar to the asexual morph of *C.aquaticum*, *C.duplumascospora*, *C.nanningense*, and *C.thailandense*. They all have monoblastic, holoblastic, conidiogenous cells and mostly irregular, brown, clathrate, muriform conidia (Liu et al. 2012). However, *C.baiseense* can be easily distinguished from *C.aquaticum*, *C.duplumascospora*, *C.nanningense*, and *C.thailandense* by its conidial septa number (6–12-transverse septa, 4–10-longitudinal septa in *C.aquaticum*; 2–4-transversely septate, 1–3-longitudinally septate in *C.duplumascospora*; 0–1 transversely septa, 0–4 longitudinal septa in *C.nanningense*; 4–8-transverse septa, 4–6-longitudinal septa in *C.thailandense* vs 0–2 transversely septa, 0–8 longitudinal septa in *C.baiseense*) and conidial size (15.5–35 × 11–26.5 μm in *C.duplumascosporum*, 45–70 × 20–57 μm in *C.aquaticum*, 25–45 × 17–33 μm in *C.thailandense*, 11–21 × 9–21 μm in *C.nanningense* vs 21 × 35–7 ×12 μm in *C.baiseense*) (Liu et al 2012; Zhang et al. 2017; Phookamsak et al. 2019). Phylogenetic reconstructions based on SSU+ITS+LSU+RBP2 sequences shows that authentic *C.baiseense* form independent monophyletic groups, well separated from *C.aquaticum*, *C.duplumascospora*, *C.nanningense*, and *C.thailandense*, respectively. A comparison of ITS sequence shows that *C.baiseense* differs from *C.aquaticum*, *C.duplumascospora*, *C.nanningense*, and *C.thailandense* in 26 bp, 24 bp, 18 bp, and 24 bp, respectively. According to the guidlines in Jeewon and Hyde (2016), we introduce *C.nanningense* as a new species.

#### Conlarium sacchari

Taxon classificationFungiAnnulatascalesAnnulatascaceae

L.Xie, Y.L.Chen & B.Liu sp. nov.

MB821681

Figure 3

##### Etymology.

The epithet “*sacchari*” refers to the habitat where first collected.

##### Type.

CHINA. Guangxi: Chongzuo City, Daxin County, Lanxu Village. 22°44'46"N, 107°15'15"E, 241 m alt., in sugarcane rhizosphere, 8 July 2015, Y.L. Chen and L.P. Qin, DX4 (HMAS 247299, holotype) deposited in Microbiology Research Institute, Guangxi Academy of Agricultural Science.

##### Description.

Colony reached 15 mm diameter on medium after 2 weeks at 28 °C, greywhite to grey, circular, flat growth, less aerial hyphae, regular edge of colony. Hyphae light yellow to yellow-brown, septate. Conidiophores yellow-brown, mostly stubby, 0–2-branched, 0–6-septate, straight or flexuous, 3–30×2–4 μm (10 ± 7 × 3 ± 1 μm, *n* = 43). Conidiogenous cells determinate, doliiform, yellow-brown to brown, 4–12 × 2–7 μm (7 ± 2 × 5 ± 1 μm, *n* = 52). Conidia yellow-brown to brown, muriform, irregularly globose or subglobose, smooth, constricted at the separation, 0–1 transversely septa, 0–4 longitudinal septa, 14–19×13–22 μm (17 ± 3 × 16 ± 2 μm, *n* = 20). **Sexual morph**: undetermined.

**Figure 3.**
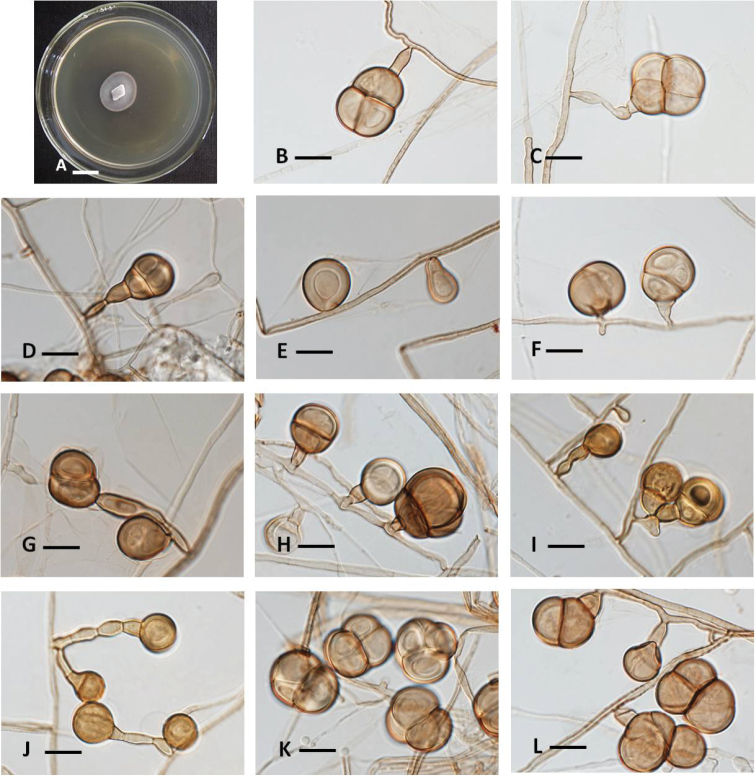
The new species *Conlariumsacchari* (holotype, HMAS 247299). **A** Colony morphology **B–L** Conidiophores, conidiogenous cells and conidia. Scale bars: 10 mm (**A**); 10 μm (**B–L**).

##### Habitat and distribution.

In sugarcane rhizosphere soil of southern China.

##### Other specimens examined.

CHINA. Guangxi: Nanning City, Long’an County, Natong Town. 23°4'48"N, 107°47'31"E, 128 m alt., in sugarcane rhizosphere, 11 Sep. 2015, Y.L. Chen and L.P. Qin, LA3 (HMAS 247300). Nanning City, Suxu town. 23°34'42"N, 108°8'30"E, 325 m alt., in sugarcane rhizosphere, 11 Feb. 2011, L. Xie, NN1 (HMAS 247301).

##### Notes.

*Conlariumsacchari* is similar to the asexual morph of *C.aquaticum*, *C.baiseense*, *C.duplumascospora*, *C.nanningense*, and *C.thailandense*. They all have monoblastic, holoblastic, conidiogenous cells and mostly irregular, brown, clathrate, muriform conidia (Liu et al. 2012). However, *Conlariumsacchari* can be easily distinguished from *C.aquaticum*, *C.duplumascospora*, *C.nanningense*, and *C.thailandense* by its less number of conidial septa (6–12-transverse septa, 4–10-longitudinal septa in *C.aquaticum*; 2–5 transversely septa, 0–2 longitudinal septa in *C.baiseense*, 2–4-transversely septate, 1–3-longitudinally septate in *C.duplumascospora*; 0–1 transversely septa, 0–4 longitudinal septa in *C.nanningense*; 4–8-transverse septa, 4–6-longitudinal septa in *C.thailandense* vs. 0–1 transversely septa, 0–3 longitudinal septa in *C.sacchari*) (Liu et al 2012; Zhang et al. 2017; Phookamsak et al. 2019). Phylogenetic reconstructions based on SSU+ITS+LSU+RBP2 sequences shows that authentic *C.sacchari* formed independent monophyletic groups which are well separated from *C.aquaticum*, *C.baiseense*, *C.duplumascospora*, *C.nanningense*, and *C.thailandense*, respectively. A comparion of ITS sequence shows that *C.sacchari* differ from *C.aquaticum*, *C.baiseense*, *C.duplumascospora*, *C.nanningense*, and *C.thailandense* in 21 bp, 24 bp, 21 bp, 18 bp, and 16 bp, resectively. Therefore, we introduce *C.sacchari* as a new species, following the guidelines of Jeewon and Hyde (2016).

### Phylogenetic analysis

**Figure 4.**
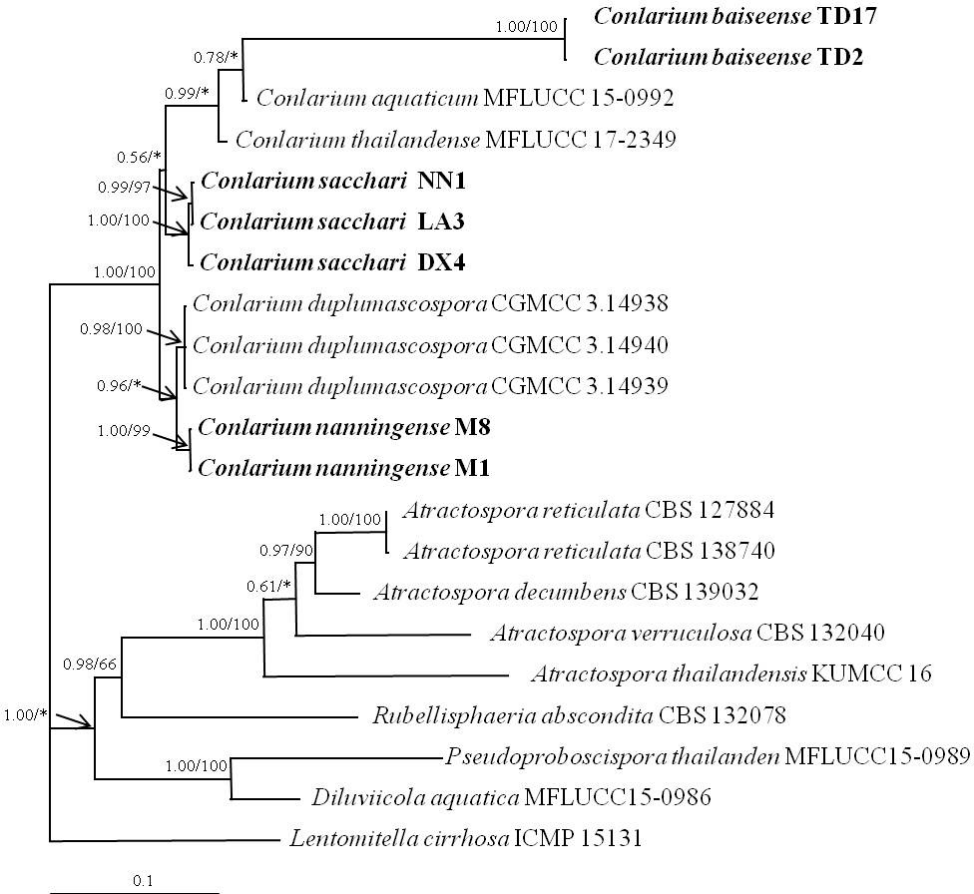
Bayesian tree based on the combined SSU+ITS+LSU+RBP2 sequences of *Conlarium* species and related families. *Lentomitellacirrhosa* was designated as outgroups. The numbers at each branch point represented Bayesian posterior probabilities (left) and percentage bootstrap support calculated from 1,000 replicates (right). *indicates lack of support or support less than 50 % for a particular clade. New species proposed are in bold. Bar 0.1 expected changes per site.

To determine the phylogenetic positions of the three new species, *C.baiseense*, *C.nanningense* and *C.sacchari*, all available SSU, ITS, LSU, and RBP2 sequences of *Conlarium* species and related genera in GenBank were downloaded (Table 1). A combined SSU+ITS+LSU+RBP2 dataset of *C.baiseense*, *C.nanningense*, and *C.sacchari*, six isolates from Atractosporaceae, two taxa from Pseudoproboscisporaceae, and *Lentomitellacirrhosa* as the outgroup, were included in the phylogenetic analysis. In the alignment of the 21 sequences (SSU+ITS+LSU+RBP2), the data matrix comprised 3293 characters. The alignment dataset was performed using the MrBayes program, applied with SYM+I+G model selected by MrModeltest as the best-fit model. The prior probability density is a flat Dirichlet (all values are 278 1.0) for both Revmatpr and Statefreqpr as default settings. A Bayesian tree with posterior probability (BPP) and bootstrap values at branches is shown in Figure 4. In the phylogenetic tree, *C.baiseense* and *C.sacchari* formed a separate clade with 1.00 support of BPP and 100% support of NJ, *C.nanningense* formed a clade with *C.duplumascospora* with 0.96 support of BPP, and three of the new species were clearly separated from other *Conlarium* species.

## Discussion

The genus *Conlarium* comprises three species, *C.duplumascosporum*, *C.thailandense*, and a hyphomycetous asexualmorph taxon, *C.aquaticum*. They have subglobose or irregular, brown, clathrate, muriform conidia (Zhang et al. 2017). The taxonomy of *Conlarium* is mainly based on the morphological characteristics of gregarious ascomata (Liu et al. 2012). However, the ascomata of *C.aquaticum*, *C.thailandense*, *C.sacchari*, *C.baiseense*, and *C.nanningense* was not observed on the medium. The new species introduced in this paper resemble the asexual morph of *Conlarium* in having muriform conidia. They can be distinguished by the fewer number of septa, as compared with *C.duplumascosporum*, *C.aquaticum*, and *C.thailandense* (Liu et al 2012; Zhang et al. 2017; Phookamsak et al. 2019). *Conlariumbaiseense* can be distinguished from other species by its columnar conidia (more transverse and less longitudinal septa) present. *Conlariumsacchari* is characterized by simple conidia (fewer septa) and its longer branched conidiophore. Phylogenetic reconstructions based on SSU+ITS+LSU+RBP2 sequences show that the three new species form independent monophyletic groups and are well separated from *C.duplumascophora*, *C.thailandense*, and *C.aquaticum*; this further supports the erection of these three new species. *Conlariumduplumascophora*, *C.thailandense*, and *C.aquaticum* were from wood samples. Our three new species present as dark spetate endophytes from sugarcane rhizosphere and can be symbiotic with sugarcane. The new species extend the habitat of *Conlarium* from wood to soil.

## Supplementary Material

XML Treatment for Conlarium nanningense

XML Treatment for Conlarium baiseense

XML Treatment for Conlarium sacchari
